# GM-CSF targeted immunomodulation affects host response to *M*. *tuberculosis* infection

**DOI:** 10.1038/s41598-018-26984-3

**Published:** 2018-06-05

**Authors:** Sulayman Benmerzoug, Fabio Vitarelli Marinho, Stéphanie Rose, Claire Mackowiak, David Gosset, Delphine Sedda, Emeline Poisson, Catherine Uyttenhove, Jacques Van Snick, Muazzam Jacobs, Irene Garcia, Bernhard Ryffel, Valerie F. J. Quesniaux

**Affiliations:** 1 CNRS, UMR7355 Orleans, France; 2 Experimental and Molecular Immunology and Neurogenetics (INEM), University of Orleans, Orleans, France; 3 Department of Biochemistry and Immunology, Institute of Biological Sciences, Federal University of Minas Gerais, Belo Horizonte-Minas, Gerais Brazil; 4 P@CYFIC Plateform, Center for Molecular Biophysics, CNRS UPR4301 Orleans, France; 5 Ludwig Cancer Research, Brussels, Belgium; 6 Division of Immunology, Department of Pathology and the Institute of Infectious Disease and Molecular Medicine, Faculty of Health Sciences, University of Cape Town, Cape Town, South Africa; 7 National Health Laboratory Service, Sandringham, Johannesburg, South Africa; 8 Immunology of Infectious Disease Research Unit, South African Medical Research Council, Cape Town, South Africa; 9 Department of Pathology and Immunology, Centre Medical Universitaire (CMU), Faculty of Medicine, University of Geneva, Geneva, 1211 Switzerland

## Abstract

Host directed immunomodulation represents potential new adjuvant therapies in infectious diseases such as tuberculosis. Major cytokines like TNFα exert a multifold role in host control of mycobacterial infections. GM-CSF and its receptor are over-expressed during acute *M*. *tuberculosis* infection and we asked how GM-CSF neutralization might affect host response, both in immunocompetent and in immunocompromised TNFα-deficient mice. GM-CSF neutralizing antibodies, at a dose effectively preventing acute lung inflammation, did not affect *M*. *tuberculosis* bacterial burden, but increased the number of granuloma in wild-type mice. We next assessed whether GM-CSF neutralization might affect the control of *M*. *tuberculosis* by isoniazid/rifampicin chemotherapy. GM-CSF neutralization compromised the bacterial control under sub-optimal isoniazid/rifampicin treatment in TNFα-deficient mice, leading to exacerbated lung inflammation with necrotic granulomatous structures and high numbers of intracellular *M*. *tuberculosis* bacilli. *In vitro*, GM-CSF neutralization promoted M2 anti-inflammatory phenotype in *M*. *bovis* BCG infected macrophages, with reduced mycobactericidal NO production and higher intracellular *M*. *bovis* BCG burden. Thus, GM-CSF pathway overexpression during acute *M*. *tuberculosis* infection contributes to an efficient M1 response, and interfering with GM-CSF pathway in the course of infection may impair the host inflammatory response against *M*. *tuberculosis*.

## Introduction

Tuberculosis (TB) remains a major global health problem with the emergence and spreading of new drug resistant strains, including in ‘developed’ countries^1^. New approaches, such as host-directed therapies (HDT) are being developed to improve the control of *Mycobacterium tuberculosis* infection^2–4^. Indeed, although one-third of the global population is considered to be infected by *M*. *tuberculosis*, only 5% to 10% will develop active disease. This indicates that a healthy host immune system effectively controls the infection, although mycobacteria remain present for years in a very slow or non-replicating dormant state^5^. Host immune responses involved in controlling *M*. *tuberculosis* infection include innate and adaptive immune responses, with multiple key players including macrophages, T cells, and mediators such as tumor necrosis factor alpha (TNFα), IFNγ, IL-1, IL-12p40, reactive oxygen and nitrogen intermediates^6–10^. In front of multidrug resistant *M*. *tuberculosis* strains, allying host immunomodulation with chemotherapy could help to control the pathogen and limit dissemination^11,12^. TNFα is essential for granuloma formation and maintenance, and experimental host directed adjunctive therapies combining TNFα neutralization with chemotherapy reported enhanced *M*. *tuberculosis* bacterial clearance^13–15^. However, neutralizing TNF is a risky measure to take in *M*. *tuberculosis* infected individuals. Here we hypothesized that GM-CSF immunomodulation could contribute to host defense against *M*. *tuberculosis*.

Indeed, a new, pivotal role of GM-CSF at the T cell - myeloid cell interface has recently emerged, and targeting the crosstalk between T cells and myeloid cells through GM-CSF seems promising in immunopathology and chronic tissue inflammation^16^. Beside its role in homeostasis, GM-CSF is considered as a key mediator in tissue inflammation, notably in chronic obstructive pulmonary disease (COPD), allergy and asthma. Neutralization of GM-CSF or its receptors are in development for indications including cancer or severe inflammatory diseases such as rheumatoid arthritis^17,18^. However, the role of GM-CSF during bacterial infections like TB is poorly understood. GM-CSF is induced after *in vitro M*. *tuberculosis* infection of THP-1 cells via PI3-K/MEK1/p38 MAPK signaling pathway^19^. Additionally, GM-CSF contributes to proinflammatory macrophage M1 polarization^20^. Indeed, GM-CSF induces monocyte and macrophage production of IL-6, IL-8, G-CSF, M-CSF, TNFα and IL-1β, although less potently than LPS^20^. On the other hand, GM-CSF seemed to control TNFα and IFNγ expression, since lung cells from GM-CSF deficient mice produced less TNFα and IFNγ after exposure to *M*. *tuberculosis* culture filtrate proteins than wild-type cells^21^.

It was proposed that GM-CSF controls granuloma formation in *M*. *tuberculosis* infection since GM-CSF deficient mice exhibited granuloma disruption^22^. However, while GM-CSF is essential for maintaining the ability of pulmonary alveolar macrophages to clear surfactant lipids and proteins from the lung surface, as evidenced in GM-CSF deficient mice^23^, it is uncertain whether GM-CSF has a direct effect on granuloma formation. Specific GM-CSF expression either in lung epithelial cells or in the hematopoietic compartment partially restored the control of the *M*. *tuberculosis* infection by GM-CSF deficient mice^22,24^.

Here, we show that GM-CSF and its receptor are overexpressed in the lung during acute *M*. *tuberculosis* infection *in vivo* and we tested how neutralizing pharmacologically GM-CSF host response might affect lung inflammation and granuloma formation, and interfere with the action of antibiotic treatment in *M*. *tuberculosis* infection. GM-CSF neutralizing antibodies that efficiently prevented sterile lung inflammation had no significant effect on bacterial burden and host response to *M*. *tuberculosis* infection in wild-type mice, while promoting granuloma formation. We then tested GM-CSF neutralization under sub-optimal antibiotic chemotherapy in TNFα-deficient mice. We propose that GM-CSF overexpression during acute *M*. *tuberculosis* infection contributes to an efficient M1 response, and neutralizing GM-CSF might be detrimental to the host inflammatory response to *M*. *tuberculosis* infection.

## Results

### Increased GM-CSF expression after *in vivo* airway *M*. *tuberculosis* infection

**Figure 1:**
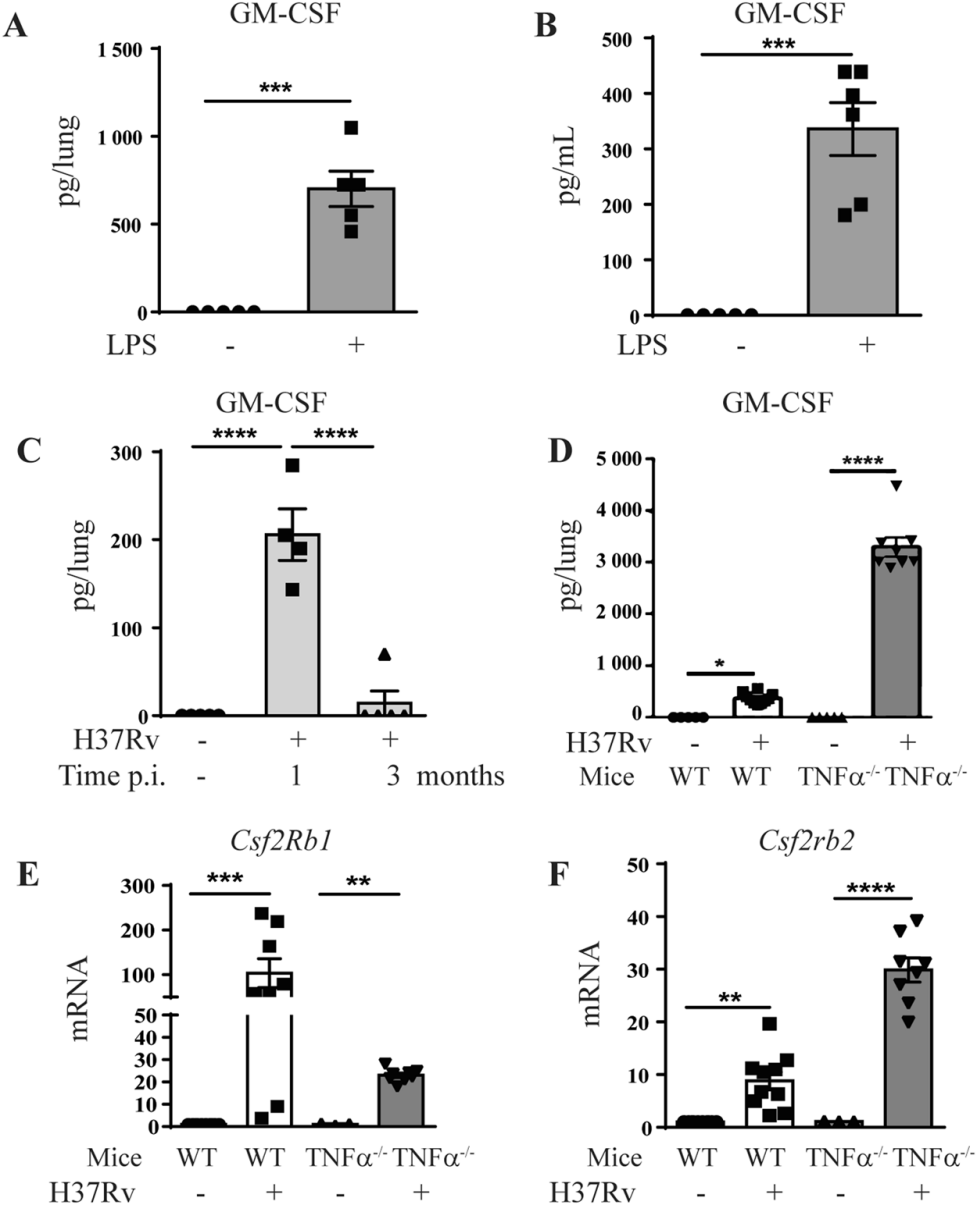
Increased pulmonary GM-CSF after acute airway inflammation and *M*. *tuberculosis* infection *in vivo*. WT mice were challenged intranasally with saline or LPS (1 µg/mouse) and GM-CSF levels in lung (**A**) and bronchoalveolar lavage (BAL) fluid (**B**) were measured after 24 h by ELISA. Pulmonary concentration of GM-CSF protein was measured 1 and 3 months (**C**) after *in vivo M*. *tuberculosis* infection (1000 ± 200 CFU/mouse i.n.) in WT mice, of after 1 month in WT and TNFα^−/−^ mice (**D**). Data are representative of two independent experiments and are expressed as mean ± SEM (n = 5–8 mice per group). The expression of GM-CSF receptor β subunits genes *Csf2rb1* (**E**) and of *Csfrb2* (**F**) in the lungs 4 weeks post-*M*. *tuberculosis* infection in WT and in TNFα^−/−^ mice was analyzed by microarray^50^. Each group of infected mice has been statistically compared to the uninfected mice of the same phenotype. ****p < 0.0001, ***p < 0.001, **p < 0.01, *p < 0.05.

We first verified that GM-CSF is regulated in the airways *in vivo* after inflammatory challenge or *M*. *tuberculosis* infection. Indeed, GM-CSF levels are increased in both bronchoalveolar lavage fluid (BAL) and lungs 24 h after LPS exposure (1 µg LPS i.t. or i.n.; Fig. 1A,B), as expected^25^, and in the lungs 1 month post *M*. *tuberculosis* infection in WT mice, although this was transient and essentially no GM-CSF was detected 3 months post-infection (Fig. 1C). Interestingly, there was also a strong upregulation of GM-CSFR β subunit genes (*Csf2rb* and *Csf2rb2*) in the lungs 28 days post- infection (Fig. 1E,F) in WT mice. Immunocompromised individuals are extremely susceptible to mycobacterial infections, and we examined here the response of TNFα-deficient mice as a model of immunodeficient mice. The production of GM-CSF and the upregulation GM-CSFR β subunit gene *Csf2rb2* were exacerbated in the lung of highly susceptible TNFα-deficient mice, 1 month post-*M*. *tuberculosis* infection (Fig. 1D,F). Thus, both GM-CSF and its receptor are over-expressed during acute *M*. *tuberculosis* infection.

### GM-CSF blockade inhibits cell recruitment after acute lung inflammation

**Figure 2:**
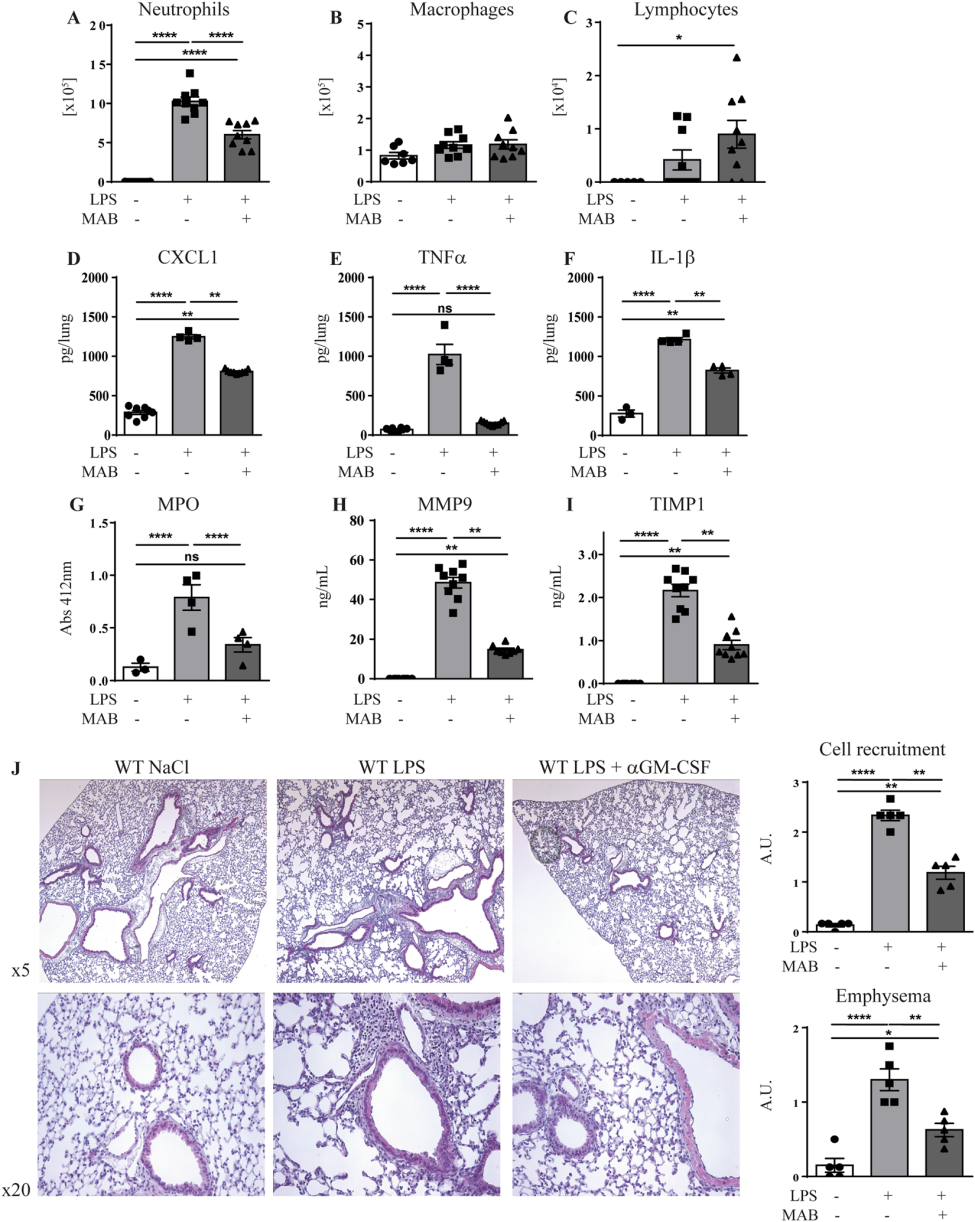
GM-CSF neutralization reduces inflammatory cell recruitment to the airways. WT mice were untreated or pre-treated with anti GM-CSF neutralizing antibody (Clone B2.6, 200 µg per mouse i.p.) 1 hour prior to LPS exposure (1 µg/mouse i.n.). Neutrophils (**A**), monocytes (**B**) and lymphocytes (**C**) recruitment were quantified in the BAL fluid after 24 h. The lung levels of CXCL1 (**D**), TNFα (**E**), IL-1β (**F**), Myeloperoxidase activity (**G**) and BAL levels of MMP9 (**H**) and TIMP1 (**I**) were measured. Lungs tissue was fixed in 4% buffered formaldehyde and HE staining is shown with a scoring of inflammatory cell recruitment and emphysema (**J**). Data are from two independent experiments (n = 4–9 mice per group) and are expressed as mean ± SEM. Statistical comparisons are presented between groups as indicated. ****p < 0.0001, **p < 0.01, *p < 0.05; ns, p > 0.05.

Tuberculous granuloma comprising macrophages, dendritic cells, lymphocytes and neutrophils are keys for the host response to *M*. *tuberculosis*^26,27^. We next assessed the contribution of GM-CSF in inflammatory cell recruitment to the lungs, by using anti-murine GM-CSF neutralizing antibodies. We first verified that anti GM-CSF neutralizing antibodies impairs acute lung inflammation and inflammatory cell recruitment in the airways after LPS exposure (1 µg/mouse i.n. and i.t). Administration of anti GM-CSF antibodies (200 µg per mouse i.p.) strongly reduced neutrophil recruitment in the bronchoalveolar lavage (BAL; Fig. 2A), as reported^28,29^, while lymphocytes were slightly increased and macrophage counts were not affected (Fig. 2B,C). GM-CSF neutralization also reduced lung CXCL1, TNFα, IL-1β and MPO response to LPS exposure (Fig. 2D–G), together with the markers of tissue inflammation MMP9 and TIMP1 in BAL (Fig. 2H,I). Histologically, GM-CSF neutralization impaired inflammatory cell recruitment and reduced emphysema after airway inflammatory challenge (Fig. 2J). Thus, systemic GM-CSF neutralization prevented acute lung inflammation, proinflammatory cytokine and chemokine release, and neutrophil recruitment in the airways.

### Limited effect of GM-CSF neutralization on acute *M*. *tuberculosis* infection

**Figure 3:**
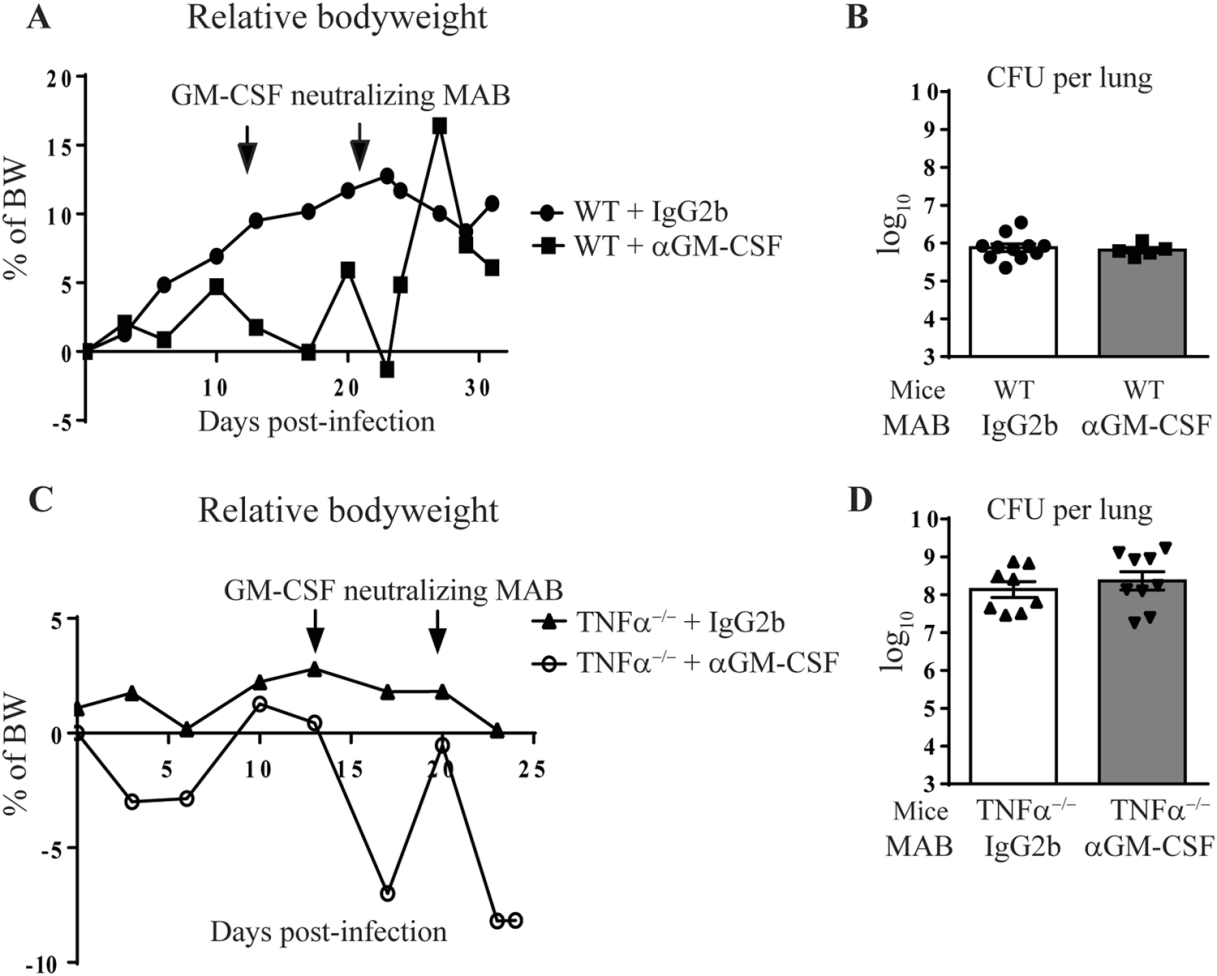
GM-CSF neutralization during *M*. *tuberculosis* infection. WT or TNFα^−/−^ mice infected with *M*. *tuberculosis* H37Rv (1000 ± 200 CFU/mouse i.n.) received either GM-CSF neutralizing MAB (Clone A7.39, 200 µg per mouse i.p.) or IgG2b isotype control on day 14 and 21 post-infection and relative body weight gain was recorded (**A**,**C**). Bacterial burden was determined in the lungs on day 26 for TNFα^−/−^ mice and on day 32 for WT mice (**B**,**D**). Data are from two independent experiments (n = 5–9 mice per group) and are expressed as mean values ± SEM. No statistical difference between the groups were observed (p > 0.05).

In view of the dual role of the inflammatory response in host control of tuberculosis, both deleterious but also indispensable for bacterial control^12^, we next asked how GM-CSF neutralization might affect the host response to *M*. *tuberculosis*. We addressed this question in wild-type C57Bl/6 mice, largely resistant to *M*. *tuberculosis* infection alike humans, and in susceptible TNFα^−/−^ mice^30^, more representative of immunocompromised conditions. GM-CSF neutralizing monoclonal antibodies (MAB) or IgG2b isotype control were administered (200 µg i.p.) on day 14 and 21 after infection with *M*. *tuberculosis* H37Rv (1000 ± 200 CFU/mouse i.n.). After a transient decrease in bodyweight following anti GM-CSF MAB administration, the susceptible TNFα^−/−^ mice showed a strong deterioration of their general status and had to be killed by day 26 ± 2, while WT mice were less affected (Fig. 3A,C). Indeed, lung bacterial loads were controlled in WT mice and were not affected by anti GM-CSF administration, as seen on day 32 post infection (Fig. 3B). TNFα^−/−^ mice had 2 log_10_ higher pulmonary bacterial loads than WT mice on day 26, which were not further affected by the anti GM-CSF treatment (Fig. 3D). Thus, administration of GM-CSF neutralizing antibodies had no impact on bacterial control in the lung of both resistant WT and susceptible TNFα^−/−^ mice, after *M*. *tuberculosis* infection.

### Effect of GM-CSF neutralization on *M*. *tuberculosis* induced lung inflammation

**Figure 4:**
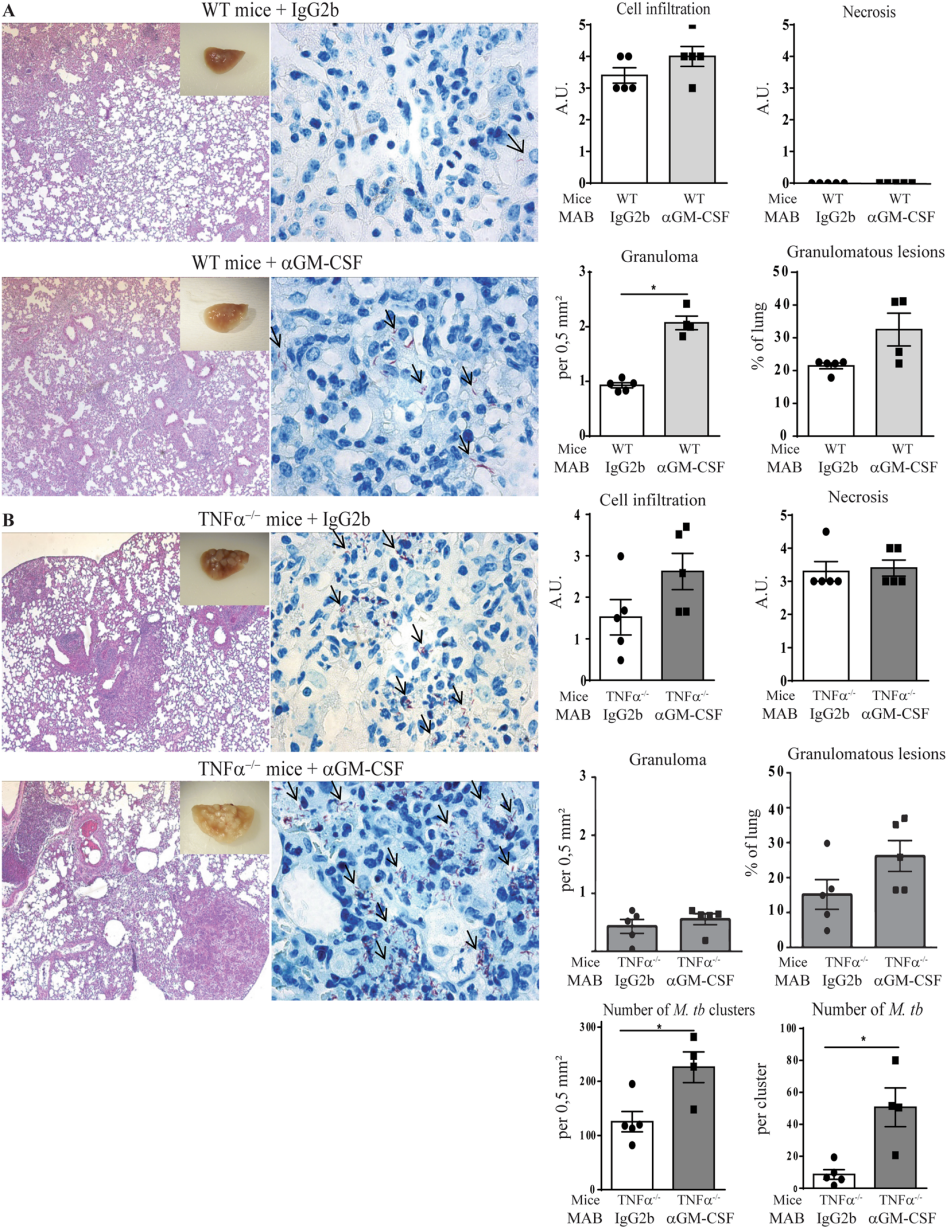
Effect of GM-CSF blockade on granuloma integrity and bacterial burden. WT (**A**) or TNFα^−/−^ mice (**B**) infected with *M*. *tuberculosis* H37Rv received either GM-CSF neutralizing MAB or IgG2b isotype control on day 14 and 21 post-infection, as in Fig. 3. Lungs from infected mice were fixed, and HE (Left panels) and ZN (Middle panels) staining performed at day 26 for TNFα^−/−^ mice and day 32 for WT mice post-infection. Black arrows point to acid-alcohol resistant bacilli. A scoring of cell infiltration and necrosis is shown (Right panels). The number of granuloma per mm² and surface of granulomatous lesions (expressed as %) were quantified on digitalized sections. Data are expressed as mean ± SEM. (n = 4–5 mice per group). Statistically significant comparisons are presented. *p < 0.05.

We next assessed the effect of GM-CSF neutralization on the lung inflammatory response, cell recruitment, and granuloma formation one month after *M*. *tuberculosis* infection. Macroscopically, WT mice receiving IgG2b isotype control showed few nodules that appeared larger in WT mice receiving αGM-CSF antibody (Fig. 4A). TNFα^−/−^ mice receiving isotype control displayed many diffuse nodules that were exacerbated in TNFα^−/−^ mice treated with αGM-CSF antibody (Fig. 4B). Microscopically, the number of granuloma present in the lung of WT mice was increased after GM-CSF neutralization, as compared to IgG2b isotype treated controls (Fig. 4A). TNFα deficient mice developed large confluent granulomatous lesions, associated with necrotic areas, which were increased after anti GM-CSF MAB treatment (Fig. 4B). Furthermore, Ziehl-Neelsen coloration of acid-alcohol resistant *M*. *tuberculosis* bacilli, detected very few *M*. *tuberculosis* bacilli in WT mice receiving IgG2b isotype control, that seemed more frequent in WT mice treated with αGM-CSF antibodies (Fig. 4A). TNFα deficient mice receiving isotype control harbored more *M*. *tuberculosis* bacilli in lung tissue as compared to WT, and the number of bacilli in the lesions of TNFα^−/−^ mice further increased after treatment with αGM-CSF antibodies, as estimated from the Ziehl-Neelsen staining (Fig. 4B). Although the overall lung inflammation, evaluated using relative lung weight as a surrogate marker, was slightly reduced in WT mice after anti GM-CSF MAB administration, this was not observed in TNFα^−/−^ mice (Supplementary Fig. S1). Spleen and liver relative weights were also unaffected in WT mice receiving isotype control or αGM-CSF antibodies while they were increased in susceptible TNFα^−/−^ mice after αGM-CSF antibody treatment as compared to isotype control. GM-CSF neutralization had no effect on the lung levels of IL-12p40 and IFNγ, two prominent cytokines involved in host control after *M*. *tuberculosis* infection, both in WT and TNFα deficient mice (Supplementary Fig. S1). Thus, GM-CSF neutralization increased the extent of granulomatous lesions and their bacilli content after *M*. *tuberculosis* infection.

### Effect of GM-CSF neutralization on *M*. *tuberculosis* infection control by chemotherapy in TNFα^−/−^ susceptible mice

**Figure 5:**
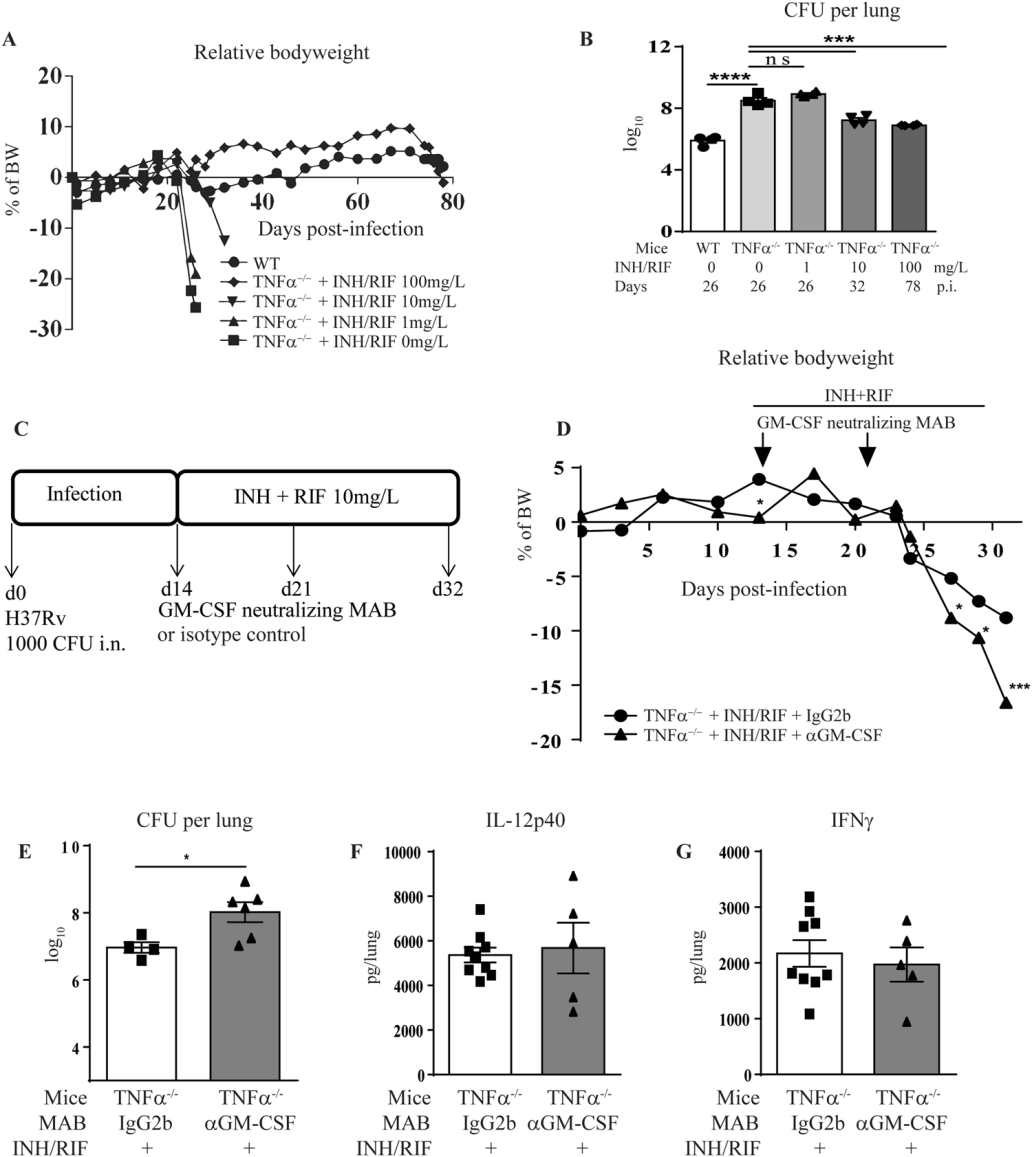
GM-CSF neutralization during treatment of *M*. *tuberculosis* infection by sub-optimal chemotherapy. WT and TNFα^−/−^ mice were infected with *M*. *tuberculosis* H37Rv (1000 ± 200 CFU/mouse i.n.) and untreated or treated with increasing doses of antibiotics (INH/RIF) in drinking water from day 14 to day 35 post-infection, as indicated. Relative body weight gain was recorded (**A**) and lung bacterial burden was measured on day 26, 32 and 78 post-infection for antibiotics doses of 1, 10 and 100 mg/L, respectively (**B**). Anti GM-CSF antibody (Clone A7.39, 200 µg per mouse i.p.) or IgG2b isotype control was combined with chemotherapy during *M*. *tuberculosis* infection. INH/RIF were administered from day 14 to day 32 post-infection and anti GM-CSF MAB administered once a week for two weeks, on day 14 and day 21 post-infection (**C**). The relative bodyweight (**D**) and lung bacterial burden were measured after INH/RIF 10 mg/L (**E**) treatments. The lung levels of IL-12p40 (**F**) and IFNγ (**G**) were measured by ELISA at day 26 post-infection after INH/RIF 10 mg/L treatment. Data expressed as mean values ± SEM (n = 4–6 mice per group). Statistical comparisons are presented as compared to isotype control treated mice, unless otherwise indicated (**B**). ****p < 0.0001, ***p < 0.001, *p < 0.05, ns, p > 0.05.

We next tested the hypothesis that alteration of granuloma organization or maintenance under GM-CSF neutralization might provide an improved access to chemotherapy. Since wild-type C57BL/6 mice are resistant to *M*. *tuberculosis* infection, surviving close to one year in normal conditions^31^, we addressed this question in a more susceptible environment. TNFα deficient mice are extremely susceptible to *M*. *tuberculosis* infection, usually succumbing within 4 weeks after *M*. *tuberculosis* exposure^32^. We first determined a suboptimal dose of the first line antibiotics isoniazid (INH) and rifampicin (RIF) bi-therapy sufficient to induce a limited protection in TNFα^−/−^ mice against *M*. *tuberculosis* infection, in order to appreciate a potential adjuvant effect of the immunotherapy. A low dose of 1 mg/L of both INH and RIF (INH/RIF) given from day 14 to 35 post-infection in drinking water was totally ineffective, with a drastic body weight loss (Fig. 5A), a high bacterial load (Fig. 5B) by day 26, similar to untreated TNFα^−/−^ mice. In contrast, a dose of 10 mg/L INH/RIF provided a partial protection, with a survival delayed by one week, reduced body weight loss and bacterial burden on day 32, while 100 mg/L INH/RIF on day 14 to 35 allowed TNFα^−/−^ mice to survive up to 78 days post-infection with controlled bacterial burden (Fig. 5A,B).

**Figure 6:**
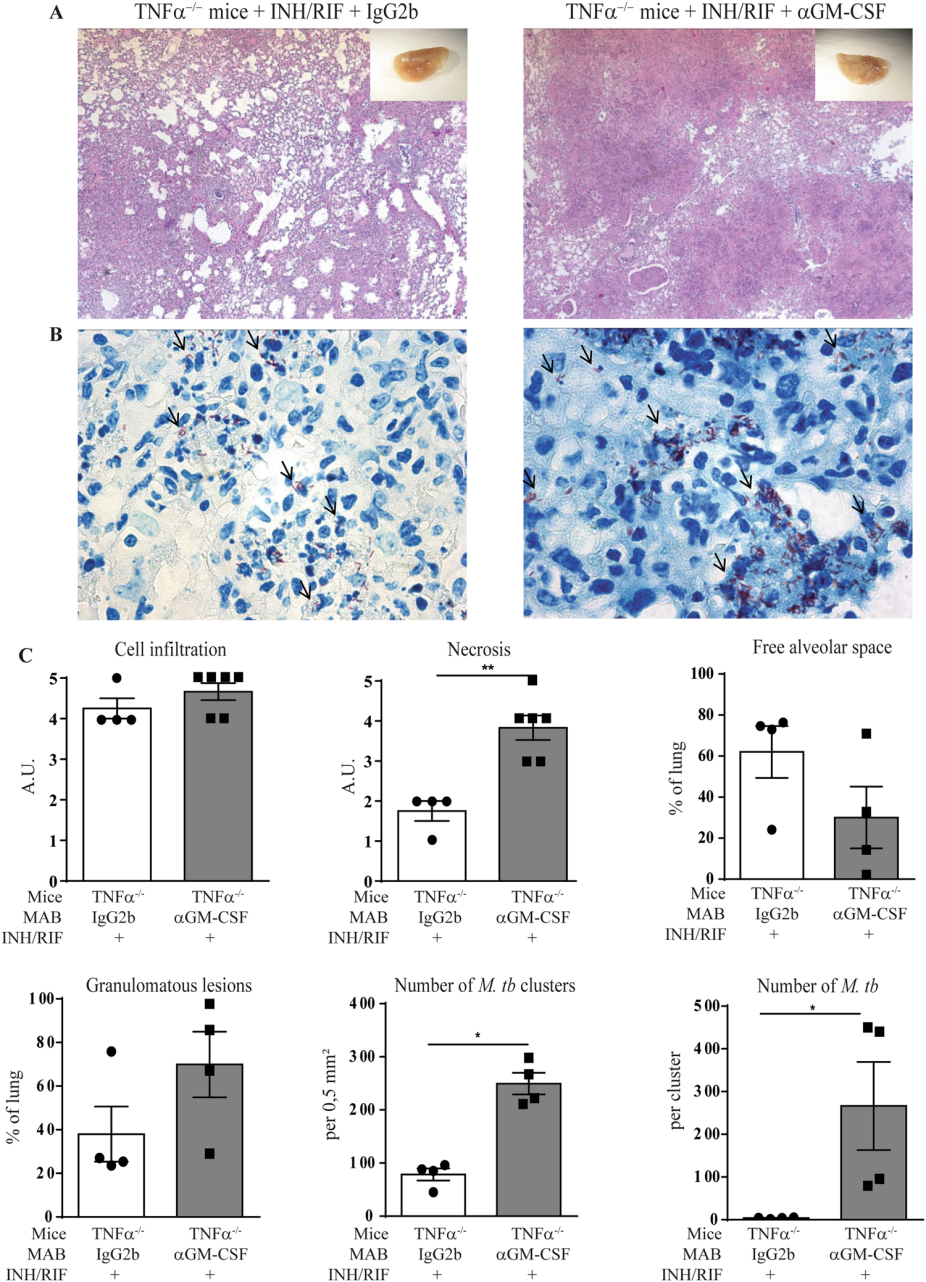
Effect of GM-CSF blockade combined with sub-optimal chemotherapy on granuloma integrity and lung bacterial burden. TNFα^−/−^ mice were infected with *M*. *tuberculosis* H37Rv (1000 ± 200 CFU/mouse i.n.), treated or not with 10 mg/L of antibiotics (INH/RIF) in drinking water from day 14 to day 32 post-infection and GM-CSF was neutralized by anti GM-CSF MAB, as indicated in Fig. 5C. On day 32 post-infection, lungs from infected mice were fixed, and stained for HE (**A**) or ZN (**B**). A scoring of cell infiltration and necrosis is shown, together with the surface of granulomatous lesions, free alveolar space, number of *M*. *tuberculosis* (*M*. *tb*) clusters per mm² and number of bacilli per cluster were quantified on digitalized sections (**C**). Blacks arrows points to acid-alcohol resistant bacilli. Data are expressed as mean ± SEM (n = 4–6 mice per group). Statistical comparisons are presented as compared to isotype control treated mice. **p < 0.01.

TNFα^−/−^ mice were infected with *M*. *tuberculosis* H37Rv, treated with INH and RIF from day 14 to 32 post-infection and received either anti GM-CSF MAB or IgG2b isotype control on day 14 and 21 post-infection (200 ug i.p.; Fig. 5C). When using a sub-optimal dose of 10 mg/L of both INH/RIF that only partially reduced the bacterial load in the TNFα^−/−^ mice (7 log_10_ CFU/lung, Fig. 5B), TNFα^−/−^ mice presented a body weight loss at day 24 post-infection (Fig. 5D) and the co-administration of anti GM-CSF MAB led to a significant increase in body weight loss (Fig. 5D) and bacterial load (Fig. 5E), as compared with isotype-treated TNFα^−/−^ mice, on day 32 post-infection. The lung levels of IL12p40 (Fig. 5F) and IFNγ (Fig. 5G) were not affected by anti GM-CSF antibodies, as compared with isotype control, in TNFα^−/−^ mice treated with INH/RIF 10 mg/L. Histologically, the sub-optimal antibiotic treatment limited the necrosis in the lung of TNFα^−/−^ mice (Fig. 6A,C), as compared to untreated TNFα^−/−^ mice (Fig. 4B). Co-administration of anti GM-CSF antibodies in antibiotic treated TNFα^−/−^ mice yielded a massive infiltration with larger nodule formation and extended necrosis, together with a trend towards decreased free alveolar space and increased granulomatous lesions (Fig. 6A,C). Very interestingly, clusters of *M*. *tuberculosis* bacilli were visible in TNFα^−/−^ mice treated with INH/RIF and their number increased after anti GM-CSF antibodies treatment, as compared with isotype control (Fig. 6B). We then verified the effect of anti GM-CSF treatment under a more effective antibacterial chemotherapy (Supplementary Fig. S2). When using a 20 mg/L INH/RIF regimen that controlled *M*. *tuberculosis* infection and yielded a strong bacterial load reduction in TNFα^−/−^ mice (down to 2 log_10_ CFU/lung), GM-CSF neutralization did not impair bacterial control, nor pulmonary IL12p40 and IFNγ levels. Lung integrity was preserved under the 20 mg/L antibiotics dose, and anti GM-CSF antibody treatment did not induce morphological differences 32 days post-infection, although macroscopically larger nodules were observed. Thus, in susceptible TNFα^−/−^ mice GM-CSF neutralization compromised the bacterial control by sub-optimal antibiotic treatment, leading to exacerbated lung inflammation, necrosis and intracellular bacterial load, while it did not affect host response under more effective antibacterial chemotherapy.

### GM-CSF neutralization promotes an anti-inflammatory M2 macrophage response after *in vitro* mycobacterial infection

**Figure 7:**
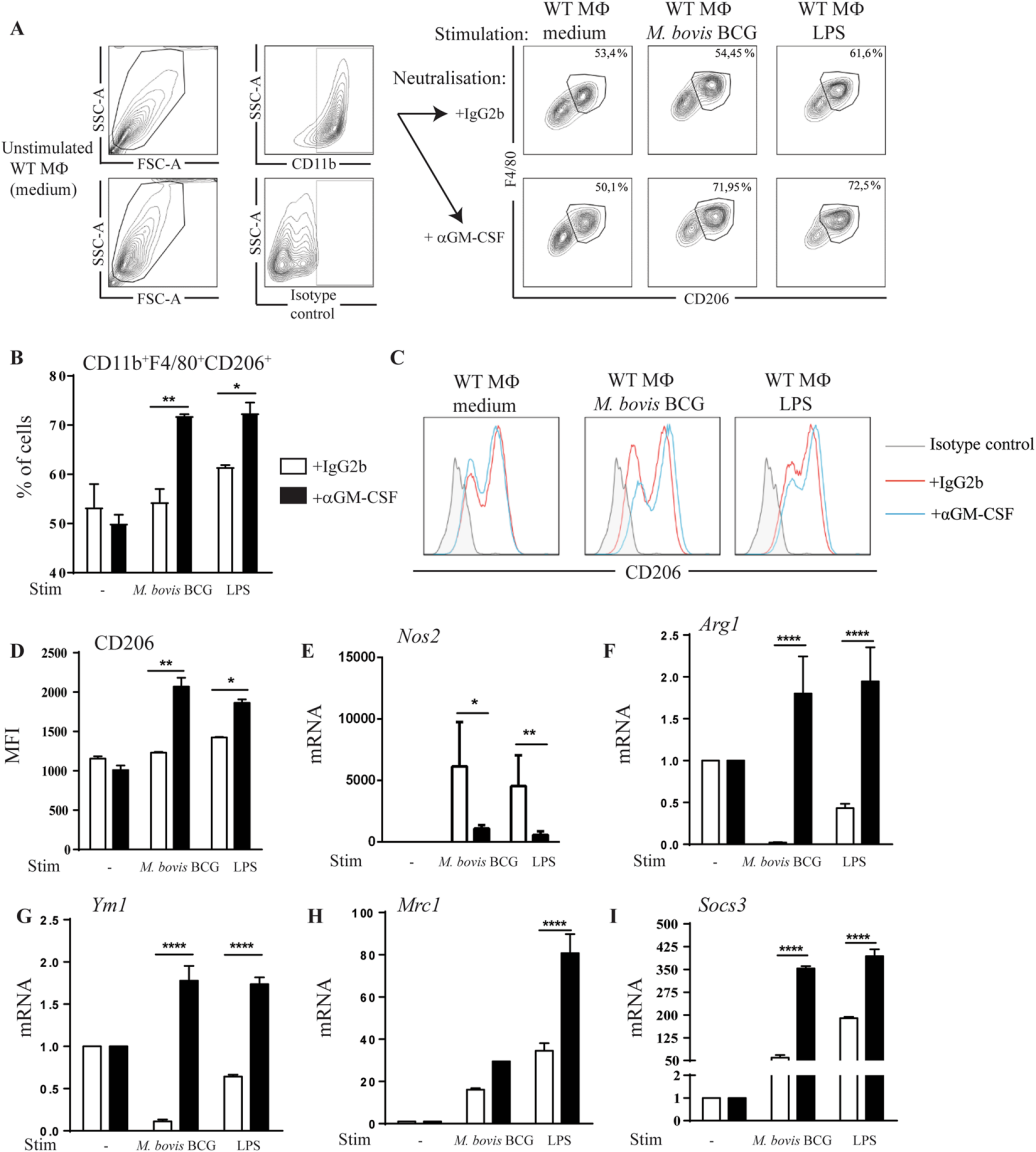
*In vitro* GM-CSF neutralization induces a switch towards M2 macrophage polarization profile after mycobacterial infection. Mannose receptor CD206 expression was determined by flow cytometry in macrophages treated with GM-CSF neutralizing MAB (Clone A7.39) or IgG2b isotype control (1 µg/mL) and either unstimulated (medium), or stimulated with *M*. *bovis* BCG (M.O.I.:2) or LPS (100 ng/mL) during 24 hours. CD11b^+^ cells were pre-gated and F4/80^+^CD206^+^ double positive cells were selected, as shown on representative dot blots (**A**), together with cell frequency (**B**). Histograms (**C**) and MFI (**D**) of BMDMs treated with anti GM-CSF MAB (blue lines) or IgG2b isotype control (red lines) and labeled with rat anti-mouse CD206-APC (clone MR5D3) antibodies are shown, as compared to isotype control (grey). Data are expressed as mean ± SD of n = 2 independent cultures. M1 and M2-related genes expression was measured in macrophages after 24 hours of stimulation. The mRNA levels of *nitric oxide synthase 2* (*Nos2*, **E**), *arginase 1* (*Arg1*, **F**)*, chitinase-like 3* (*Ym1*, **G**), CD206 *mannose receptor, C type 1* (*Mrc1*, **H**) and *suppressor of cytokine signaling 3* (*Socs3*, **I**) were analyzed by RT-qPCR. The fold change of mRNA levels, normalized to *Gapdh* level in medium, is shown. Data are from two independent experiments and are expressed as mean values ± SEM of n = 4 independent cultures. Statistical comparisons are presented as compared to isotype control treated cells. ****p < 0.0001, **p < 0.01, *p < 0.05.

GM-CSF has recently been added to the stimuli contributing to macrophage polarization towards M1 profile^20^, and we thus asked how GM-CSF neutralization might impact M1/M2 polarization *in vitro*. Macrophages express GM-CSF after *in vitro M*. *bovis BCG* infection^33^. To appreciate the effects of GM-CSF neutralization on macrophage polarization and mycobacterial control *in vitro*, bone marrow-derived macrophages were stimulated with LPS or *M*. *bovis* BCG in the presence of anti-GM-CSF antibody or IgG2b isotype control. GM-CSF neutralization led to an increase in CD11b^+^F4/80^+^CD206^+^ M2 macrophage population and in CD206^+^ MFI values, as compared to isotype control, after 24 hour stimulation with LPS or *M*. *bovis* BCG (Fig. 7A–D). Further, the overexpression of the M1 marker *Nos2* induced by LPS stimulation or *M*. *bovis* BCG infection, was reduced after GM-CSF neutralization (Fig. 7E). Conversely, GM-CSF neutralization yielded the overexpression of all M2-related genes, including *Arg1* and *Ym1* that are downregulated after LPS stimulation and *M*. *bovis* BCG infection, and including also CD206 coding gene *Mrc1* and the regulatory gene *Socs3*, that are already upregulated by LPS or *M*. *bovis* BCG (Fig. 7E–I). Thus, GM-CSF neutralization influenced M1/M2 macrophage balance in favor of M2 macrophages after *in vitro M*. *bovis* BCG infection.

### M2 macrophages induced by GM-CSF neutralization fail to produce nitric oxide culminating in intracellular persistence of mycobacteria

**Figure 8:**
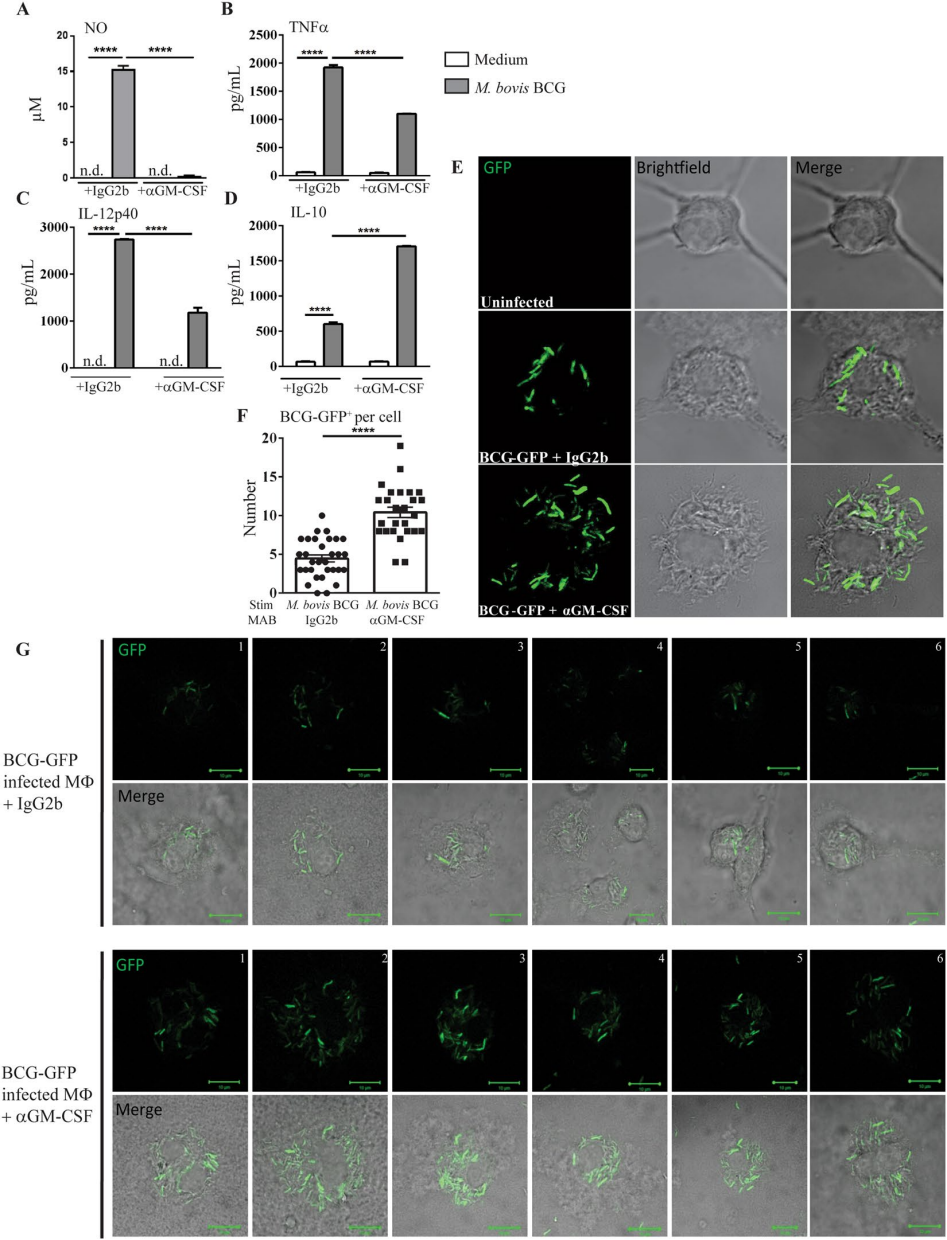
*In vitro* GM-CSF blockade is deleterious for nitric oxide production and mycobacterial killing. Macrophages treated with GM-CSF neutralizing MAB (Clone A7.39) or IgG2b isotype control (1 µg/mL) were either unstimulated (medium), or infected with *M*. *bovis* BCG-GFP (MOI:4) during 24 hours. After 24 hours, NO production was quantified in cell supernatant using Griess reaction^7^ (**A**) and TNFα (**B**), IL12p40 (**C**) and IL-10 (**D**) levels were quantified by ELISA. Infected macrophages were also fixed in paraformaldehyde 4% during 10 minutes and mounted on slides using Fluoromount® (**E**). Cells were observed in confocal microscopy at 63x magnitude. A quantification of GFP^+^ *M*. *bovis* BCG per cell is shown (**F**). Six additional fields with individual cells infected with BCG-GFP in the presence of anti GM-CSF MAB or isotype control are shown (**G**). Data are expressed as mean ± SD of n = 2 independent cultures (**A**–**D**) or n = 26–32 individual cells (**F**). Statistical comparisons are presented as compared to non-infected cells or in between groups treated with GM-CSF neutralizing MAB, as indicated. ****p < 0.0001, ND, Not Detected.

To evaluate the role of GM-CSF in mycobacterial killing, bone marrow-derived macrophages infected with *M*. *bovis* BCG-GFP were treated with anti GM-CSF antibodies or isotype control. Anti GM-CSF MAB treatment abolished the strong nitric oxide production by macrophages after *M*. *bovis* BCG-GFP *in vitro* infection, as compared to isotype control (Fig. 8A). Pro-inflammatory TNFα and IL-12p40 were also reduced in infected macrophages treated with anti GM-CSF MAB (Fig. 8B,C), while anti-inflammatory IL-10 was increased after GM-CSF neutralization (Fig. 8D). Interestingly, macrophages treated with anti GM-CSF MAB displayed an increased number of intracellular mycobacteria as compared to macrophages treated with isotype control (Fig. 8E–G). Thus, *in vitro* GM-CSF neutralization favors an anti-inflammatory profile in macrophages, abrogating the release of mycobactericidal nitric oxide, and leading to an increased intracellular presence of *M*. *bovis* BCG-GFP.

### Impact of *in vivo* GM-CSF blockade on macrophage polarization after *M*. *tuberculosis* infection

We next investigated the impact of GM-CSF neutralization on M1/M2 polarization *in vivo*. In a typical pulmonary M1 context after *in vivo* LPS administration (1 µg LPS i.t.), the upregulation of M1 related genes *Nos2* and *Il12b* was reduced in the lung after GM-CSF neutralization (Supplementary Fig. S3). In contrast, M2 expression markers *Arg1* and *Il10* were not affected by GM-CSF neutralizing antibodies. After *in vivo M*. *tuberculosis* infection, *Nos2* was overexpressed, while *Arg1* expression was unchanged in WT and TNFα^−/−^ mice treated with isotype control (Supplementary Fig. S3). In this context, GM-CSF neutralization did not affect the M1 or M2 macrophages related genes *Nos2* and *Arg1* in *M*. *tuberculosis* infected WT or TNFα^−/−^ mice, nor did it affect M2 related genes *Mrc1, Ym1* and *Socs3* in TNFα^−/−^ mice. Thus, GM-CSF neutralization reduced M1 polarization in an acute LPS-induced lung inflammation, but it did not affect *M*. *tuberculosis* induced M1 phenotype *in vivo*.

## Discussion

Although the contribution of GM-CSF in granuloma formation after *M*. *tuberculosis* infection based on granuloma disruption in GM-CSF deficient mice^22^ raised questions due to the defect of alveolar macrophage maturation and surfactant metabolism in these mice^23^, the role of GM-CSF in the control of *M*. *tuberculosis* infection is triggering new interest. Indeed, among patients with high anti GM-CSF autoantibody titers that had associated cryptococcal meningitis, one out of seven also developed *M*. *tuberculosis* infection^34^.

We document here that both GM-CSF and its receptor are overexpressed during acute *M*. *tuberculosis* infection, especially in highly susceptible, immunocompromised TNFα deficient mice. These results are in line with GM-CSF expression during *M*. *tuberculosis* infection of immunocompetent mice^24,35^. Beside cellular sources of GM-CSF such as alveolar macrophages and type II epithelial cells^22^, iNKT and γδ T cells can sustain early host response to *M*. *tuberculosis* in the absence of IFNγ^36^, and CD4^+^ T cells that take over later are sufficient to confer protection in a GM-CSF-depleted non-hematopoietic environment^24^. GM-CSF induces *M*. *tuberculosis* killing by macrophages, an activity requiring PPARγ expression^24^, however the exact mechanism for this effect is still unknown.

Given the double-edge role of the inflammatory response in host control of mycobacterial infection, indispensable to ensure bacterial control but potentially deleterious, we asked how GM-CSF neutralization might affect host response to *M*. *tuberculosis*. GM-CSF neutralizing antibodies, used at a dose that effectively prevents LPS-induced lung inflammation, exacerbated granuloma formation after *M*. *tuberculosis* infection and increased the number of granulomatous lesions containing large clusters of *M*. *tuberculosis* in the lung tissue of TNFα deficient mice, although this did not translate into overall increased numbers of CFU per lung.

Adjuvant immunotherapies under discussion for tuberculosis^11,12^ include the use of anti TNFα antibodies to alter granuloma structure and favor the access of antibiotics to the mycobacteria within the granulomatous structure^13^. Here, we thus assessed whether GM-CSF neutralization might affect *M*. *tuberculosis* control by sub-optimal chemotherapy in susceptible TNFα-deficient mice. Sub-optimal isoniazid (INH) and rifampicin (RIF) bi-therapy partially contained the infection in terms of CFU titer per lung, number of *M*. *tuberculosis* clusters and the number of *M*. *tuberculosis* per cluster, and reduced necrosis in the lung tissue. GM-CSF neutralization actually compromised the bacterial control by sub-optimal INH/RIF treatment in TNFα^−/−^ mice, leading to extended necrotic granulomatous lesions. However, under a higher dose chemotherapy that effectively reduced pulmonary bacterial load, the anti GM-CSF treatment did not compromise bacterial control. There was a transient body weight loss after GM-CSF neutralization in infected mice. Cachexia may result from systemic ‘cytokine storm’ as seen during a systemic inflammatory response syndrome. Indeed body weight loss did not seem an effect of the anti-GM-CSF MAB administration itself but occurred in conjunction with *M*. *tuberculosis* infection, as there was no body weight loss in the sterile LPS-induced lung inflammation model, nor in infected mice under INH/RIF antibiotics chemotherapy.

Macrophage polarization during *M*. *tuberculosis* infection is still a matter of debate. Indeed, M1 macrophages seem to play a key role in bacterial killing and granuloma formation while M2 macrophages were found in non-granulomatous lung tissues, inhibiting M1 macrophage effects^37^. Therefore, in tuberculosis a strong regulation of M1/M2 macrophages balance is essential for the host defense. GM-CSF was recently recognized to induce M1 polarization, culminating in monocyte and macrophage IL-6, IL-8, G-CSF, M-CSF, TNFα and IL-1β production^20^. Here, we report that macrophages produce GM-CSF after *M*. *bovis* BCG infection and hypothesized that GM-CSF may contribute to the host response to mycobacteria infection by favoring macrophage M1 polarization. We show that *in vitro* GM-CSF neutralization induced a shift towards M2 macrophages after *M*. *bovis* BCG infection. Indeed, blocking GM-CSF triggered the overproduction of anti-inflammatory IL-10, while TNFα and IL-12, pro-inflammatory cytokines which play a key role for the host-response to tuberculosis, were reduced. Although GM-CSF increased the phagocytic capacity of alveolar macrophages *in vitro*^35^, we show here that anti GM-CSF treatment increased the intracellular bacterial load in macrophages *in vitr*o but also *in vivo*. Thus, we propose that the anti-inflammatory milieu induced by GM-CSF neutralization impairs the ability of macrophages to eradicate mycobacteria. Indeed, while M2-related markers such as *Arg1* and *Ym1* genes or CD206 were overexpressed after GM-CSF neutralization, *M*. *bovis* BCG-induced M1 marker *Nos2* was reduced, leading to a deficient production of nitric oxide in macrophages infected *in vitro* in the presence of anti-GM-CSF antibodies.

We next hypothesized that GM-CSF overexpression during acute infection contributes to an efficient M1 response against *M*. *tuberculosis*. Although, *in vivo* GM-CSF MAB treatment reduced M1 polarization after LPS-induced acute lung inflammation, GM-CSF neutralization did not influence the M1/M2 balance in the context of *M*. *tuberculosis* infection. Indeed, several *in vivo* factors such as extracellular matrix composition, cell maturation as well as cell adhesion could greatly influence macrophages polarization^20^. During *M*. *tuberculosis* infection, high levels of Th1-derived IFNγ, but also TNFα and IL-12 produced by macrophages, dendritic cells and neutrophils contribute to a complex milieu favoring M1 macrophage polarization^32^. Thus, although GM-CSF played a prominent role for M1 macrophage polarization in an acute LPS-induced lung inflammation model, GM-CSF was not essential for M1 polarization during *M*. *tuberculosis* infection. Indeed, the complex M1 milieu was not affected by GM-CSF neutralization, with no M1/M2 switch, indicating that the main pathway inducing M1 macrophage polarization after *M*. *tuberculosis in vivo* infection is not GM-CSF-dependent but most likely IFNγ produced by Th1, NK or NKT cells^38–40^.

Anti-cytokine autoantibodies present at steady-state may cause susceptibility to infections^41^. In healthy individuals, anti-GM-CSF autoantibodies present at low levels may neutralize GM-CSF and therefore regulate GM-CSF mediated inflammation. In contrast, high levels of anti-GM-CSF autoantibodies have been associated with pulmonary alveolar proteinosis, and disseminated *Crytococcus* and *Nocardia* infections^41^. Higher levels of anti-GM-CSF autoantibodies, together with anti-IFNγ antibodies have been reported in patients with pulmonary disease due to non-tuberculous mycobacteria (NTM), relative to healthy controls, questioning whether these anti-cytokine autoantibodies might be a predisposing factor for pulmonary NTM disease^42^.

Several antibodies neutralizing GM-CSF or its receptor are in development for indications such as severe inflammatory diseases, chronic myeloid leukemia or cancer, either humanized IgG1 (leuzilumab) or fully human IgG (gimsilumab, namilumab). Mavrilimumab, a human monoclonal antibody targeting GM-CSFRα, has been tested in subjects with rheumatoid arthritis^17,18^. Our data, using anti GM-CSF antibodies in a tuberculosis infectious model may be relevant for the neutralization of GM-CSF pathway in the clinic. Indeed, we show here that murine monoclonal antibodies to GM-CSF induce a modification of granuloma integrity in the lung of immunocompromised mice infected by *M*. *tuberculosis*. The data raise concerns about the effect that such GM-CSF neutralizing therapies may have on latent tuberculosis infection in patients suffering from chronic inflammatory diseases. Indeed, recent mavrilimumab long-term safety and efficacy phase IIb studies in rheumatoid arthritis patients identified upper respiratory tract infection, including 2 cases of active pulmonary tuberculosis infection (0.22/100 PY) in patients with no active or latent tuberculosis at screening^43,44^. Thus, neutralization or adjuvant host-directed therapies using antibodies to a major cytokine such as GM-CSF might compromise the balance of host responses to mycobacterial infection and host immune system should be modulated with care in future immunotherapies.

## Materials and Methods

### Mice

C57BL/6 (B6) WT mice and TNFα deficient mice (TNFα^−/−^)^45^ were bred in our specific pathogen free animal facility at CNRS (TAAM UPS44, Orleans, France). For experiments, adult (8–12 week old) animals were kept in ventilated cages or for the infectious protocols in biological isolation safety cabinet glove boxes in a biohazard animal unit. The infected mice were monitored every day for clinical status, weighted twice weekly and were sacrificed in accordance with ethical guidelines whenever reaching an endpoint such as 20% bodyweight loss. This study was carried out in accordance with the recommendations of the French Government’s animal experiment regulations and the protocol was approved by the “Ethics Committee for Animal Experimentation of CNRS Campus Orleans” (CCO) under number CLE CCO 2015-1085 and CLE CCO 2015-1071.

### Antibodies

Mouse anti-mouse GM-CSF monoclonal antibodies (IgG1 Clone B2.6 or IgG2b Clone A7.39), obtained after immunization as described^46^, were administered (200 µg/mouse i.p.) on the indicated days. IgG1 (Clone MAB005, R&D) and IgG2b (Clone MPC-11, Bio X Cell)^47^ were used as isotype controls, respectively, as indicated.

### Acute airway inflammation

LPS from *Escherichia coli* (serotype O111:B4; Sigma, St Louis, MO, USA) diluted in saline (1 µg per mouse) or vehicle alone was applied by nasal or intra-tracheal instillation in a volume of 40 µL under light ketamine (100 mg/kg)-xylasine (10 mg/kg) anaesthesia.

Bronchoalveolar lavage (BAL) fluid was collected by canulating the trachea and washing the lungs 4 times with 0.5 mL of ice-cold PBS. After centrifugation at 1850 rpm for 10 min at 4 °C, the supernatant of the first lavage was stored at −80 °C for cytokine analysis. Cell pellets were recovered, pooled and counted. For differential counts, cell cytospins were stained with Diff-Quik Staining (Merz & Dade AG., Dudingen, Switzerland) and twice 200 cells scored.

### Infection

Aliquots of *M*. *tuberculosis* H37Rv kept frozen at −80 °C were thawed, diluted in sterile saline containing 0.05% Tween 20 and clumping disrupted by 50 repeated aspirations through 18, 20, 26 and 27 gauge needles (Omnican, Braun, Germany). Pulmonary infection with *M*. *tuberculosis* H37Rv was performed by delivering 1000 ± 200 CFU/mouse into the nasal cavities under ketamine-xylasine anesthesia as above. The bacterial load in the lungs was determined on day 1 post infection on control mice. Isoniazid (INH) and Rifampicin (RIF) were administered at the indicated concentration (1 to 100 mg/L) dissolved in drinking water from day 14 to 35 post-infection. For quantifying bacterial burden, lungs were weighted, defined aliquots homogenized in phosphate-buffered saline (PBS; Dispomix homogenizer, Medic Tools, Switzerland), and tenfold serial dilutions prepared in 0.05% Tween 20 containing 0.9% NaCl were plated in duplicates onto Middlebrook 7H11 (Difco) agar plates containing 10% OADC. After 3 weeks incubation at 37 °C colonies were enumerated and results expressed as log_10_ CFU per organ.

### Histopathological analysis

Lung tissues were fixed in 4% buffered formaldehyde overnight, paraffin-embedded, and 3 µm sections were stained with haematoxylin and eosin (HE). Lung cellular infiltration, emphysema and necrosis were quantified using a semi-quantitative score with increasing severity of changes (0–5). The area surface of pulmonary granulomatous lesions were quantified on digitalized sections using a Zeiss Axio Scan Z1 Zeiss microscope and the ZEN software. Granulomatous lesions were determined in two lobes per mouse comprising a lung surface area of 25 to 35 mm^2^ per lung section. Data are represented as percentage of granulomatous lesions corresponding to % area of lesions in mm^2^/total lung tissue in mm^2^. The number of bacterial clusters was counted in the lung lesions (0.5 mm²) and the average number of bacilli per cluster estimated in the same surface area of granulomatous lesions. Analyses were performed by two independent observers including a trained pathologist.

### Cell culture and treatments

Bone marrow cells harvested from femur of C57/BL6 wild-type mice were cultured for 10 days in Dulbecco’s modified Eagle’s medium supplemented with 2 mM L-Glutamine, 25 mM HEPES, 20% heat-inactivated horse serum, 30% L929 supernatant containing macrophage-colony stimulating factor (M-CSF), 100 U/mL penicillin and 100 µg/mL streptomycin (Gibco)^48^. Bone marrow derived macrophages (BMDM) were then harvested with cold PBS (Dulbecco), and 10^6^ and 10^5^ cells per well dispensed into 48 and 96 well plates (Corning® and Costar® Multiple Well Plates), respectively. Cells were incubated with 1 µg/mL of anti GM-CSF (Clone A7.39) or IgG2b isotype control (clone MPC-11, Bio X Cell) and stimulated with *M*. *bovis* BCG (MOI:2), *M*. *bovis* BCG-GFP (MOI:4) or LPS 100 ng/mL (Lipopolysaccharide *E*. *coli* 055:B5, Sigma-Aldrich, St Louis, MO, USA), as indicated.

For bone marrow derived dendritic cells (BMDC), bone marrow cells were cultured for 10 days in Roswell Park Memorial Institute RPMI medium supplemented with 25 mM Hepes, 1 mM sodium pyruvate, 40 µg/ml gentamycin, 50 µmol/L 2-mercaptoethanol, 10 mM L-Glutamine, 0.2% vitamins (Gibco^®^), 100 U/mL penicillin and 100 µg/mL streptomycin (Gibco), 10% decomplemented FCS and 500 µL/plate of J558 supernatants containing GM-CSF^49^. BMDC were then harvested and stimulated with *M*. *bovis* BCG (MOI:2) or 100 ng/mL LPS, as above.

### Flow cytometry

After 24 hours of stimulation, cells were washed twice in Ca^2+^/Mg^2+^-free PBS containing 3% FCS, 0.5 mM EDTA and stained following the manufacturer protocols. Rat anti-mouse CD11b-PerCp-Cy5.5 (clone M1/70), F4/80-V450 (clone BM8) and CD206-APC (clone MR5D3) were from BD Biosciences. Stained cells were washed twice as above, fixed with 1% paraformaldehyde (FACS Lysing solution, BD) and analyzed with a CANTO II analyser (Becton Dickinson). Data were processed with FlowJo software (version 7.6.5 for Windows, FlowJo LLC, Ashland, Oregon).

### Determination of cytokines and mediators

TNFα, IL-1α, IL-1β, IL-12p40, GM-CSF, CXCL1, IFNγ, MMP9, TIMP1 and MPO protein concentration in the BAL fluid, lung homogenate or cell culture supernatants were quantified by enzyme-linked immunosorbent assay (ELISA; R&D Duoset, Systems, Abingdon, UK) following the manufacturer’s instructions. Lung homogenates were centrifuged (3 min at 14,500 rpm), the supernatants sterilized by centrifugation through 0.45 µm and 0.22 µm filters (3 min at 14,500 rpm; Costar-Corning, Badhoevedorp, The Netherlands), were immediately frozen on dry ice and stored at −80 °C until determination.

### Confocal microscopy

Macrophages adhered on glass slides for 18 h at 37 °C were stimulated with *M*. *bovis* BCG-GFP (MOI:4) and with anti GM-CSF antibodies (Clone A7.39; 1 µg/mL) or IgG2b isotype control (1 µg/mL). After 24 hours, the slides were washed twice in PBS, fixed in PFA 4% for 15 min, and coverslips mounted using Fluoromount®. The cells were observed using a confocal microscope LSM510 Meta (Carl Zeiss SAS) with a Plan-Apochromat 63x/1.4 OIL objective. Images were processed using LSM510 Meta software and ImageJ.

### Transcriptome analyses

The current study included microarray data of Csf2rb, Csfrb2 and Csf2 genes present in a previously reported^50^ whole transcriptome microarray analysis of mouse lungs 4 weeks post-*M tuberculosis* infection in WT and TNFα^−/−^ mice publicly available in ArrayExpress (www.ebi.ac.uk/arrayexpress) under accession number E-MTAB-5218, www.nature.com/scientificreports/Scientific Reports|6:36923| 10.1038/srep36923. For quantitative PCR determination of mRNA expression, total RNA was isolated from lung or cells using TRI-Reagent (Sigma) and RNA integrity and quality controlled using Agilent RNA 6000 Nano kit®. Reverse transcription was performed with SuperScript®III Kit (Invitrogen) and cDNA was subjected to quantitative real-time PCR using primers for *Nos2*, *Arg1*, *Mrc1*, *Ym1* and *Socs3* (Qiagen) and GoTaq® qPCR-Master Mix (Promega). *Gapdh* expression (Qiagen) was used for normalization. Raw data were analyzed using the comparative analysis of relative expression, using ΔΔCt methods^51^.

### Statistical analysis

Statistical significance was determined with GraphPad Prism (version 5.04 for Windows, GraphPad Software, La Jolla, CA). Differences between multiple *in vivo* groups were analysed by means of one-way non-parametric ANOVA test (Kruskal-Wallis followed by Dunn’s multiple comparison test, alpha level = 0.05) and values of p ≤ 0.05 were considered significant. Two-tailed, non-parametric Mann-Whitney test was used for analysing *in vitro* results. Grubb’s test was used to exclude significant outliers.

### Data availability statement

The datasets generated during and/or analysed during the current study are available from the corresponding author on reasonable request.

## Electronic supplementary material

Supplementary Figures S1 to S3
