# Supplementary material for: GM-CSF targeted immunomodulation affects host response to M. tuberculosis infection
Source: Sci Rep. 2018 Jun 5;8:8652. doi: 10.1038/s41598-018-26984-3 (PMC5988704; doi:10.1038/s41598-018-26984-3)
Supplement: Supplementary file 1 — Supplementary Figures S1 to S3 [file 41598_2018_26984_MOESM1_ESM.docx]

**Supplementary Information**

**GM-CSF targeted immunomodulation affects host response to *M. tuberculosis* infection**

Sulayman Benmerzoug^1,2^, Fabio Vitarelli Marinho^1,3^, Stéphanie Rose^1,2^, Claire Mackowiak^1,2^, David Gosset^4^, Delphine Sedda^1,2^, Emeline Poisson ^1,2^, Catherine Uyttenhove^5^, Jacques Van Snick^5^, Muazzam Jacobs^6,7,8^, Irene Garcia^9^, Bernhard Ryffel^1,2,6^ and Valerie F. J. Quesniaux^1,2*^

**
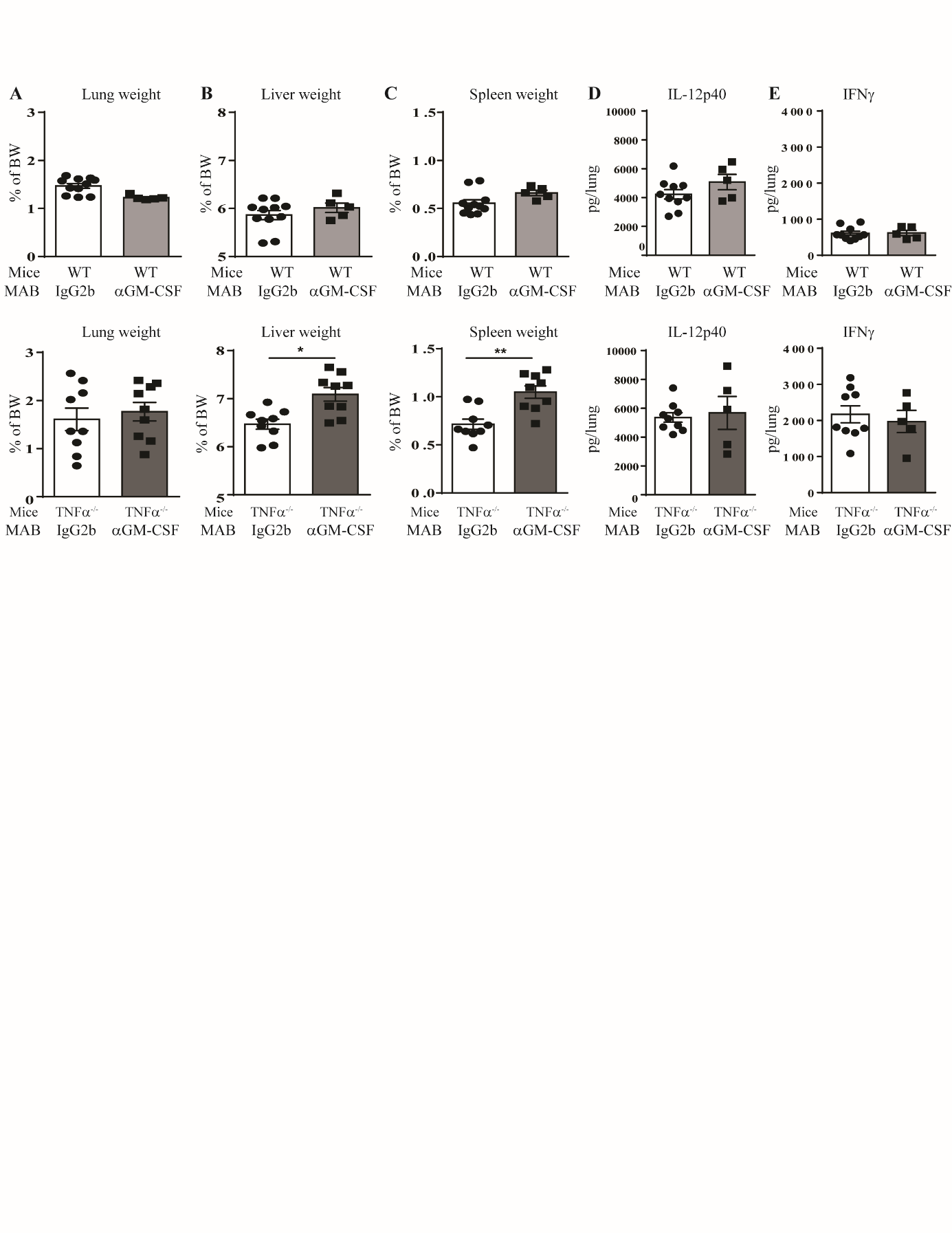
**

**Supplementary Figure 1. Inflammatory response under GM-CSF neutralization during *M. tuberculosis* infection**

WT or TNFα^-/-^ mice infected with *M. tuberculosis* H37Rv received either GM-CSF neutralizing MAB or IgG2b isotype control as in Figure 3. Relative lung (A), liver (B) and spleen (C) weights were measured 32 days post-infection for WT mice (Upper panel) and 26 days post-infection for TNFα^-/-^ mice (Lower panel). The lung levels of IL-12p40 (D) and IFNγ (E) were measured by ELISA at these time points. Data are from two independent experiments and are expressed as mean values ± SEM (n = 5-10 mice per group). Statistical comparisons are presented as compared to isotype control treated mice, as indicated. **, p < 0.01, *, p < 0.05.

**
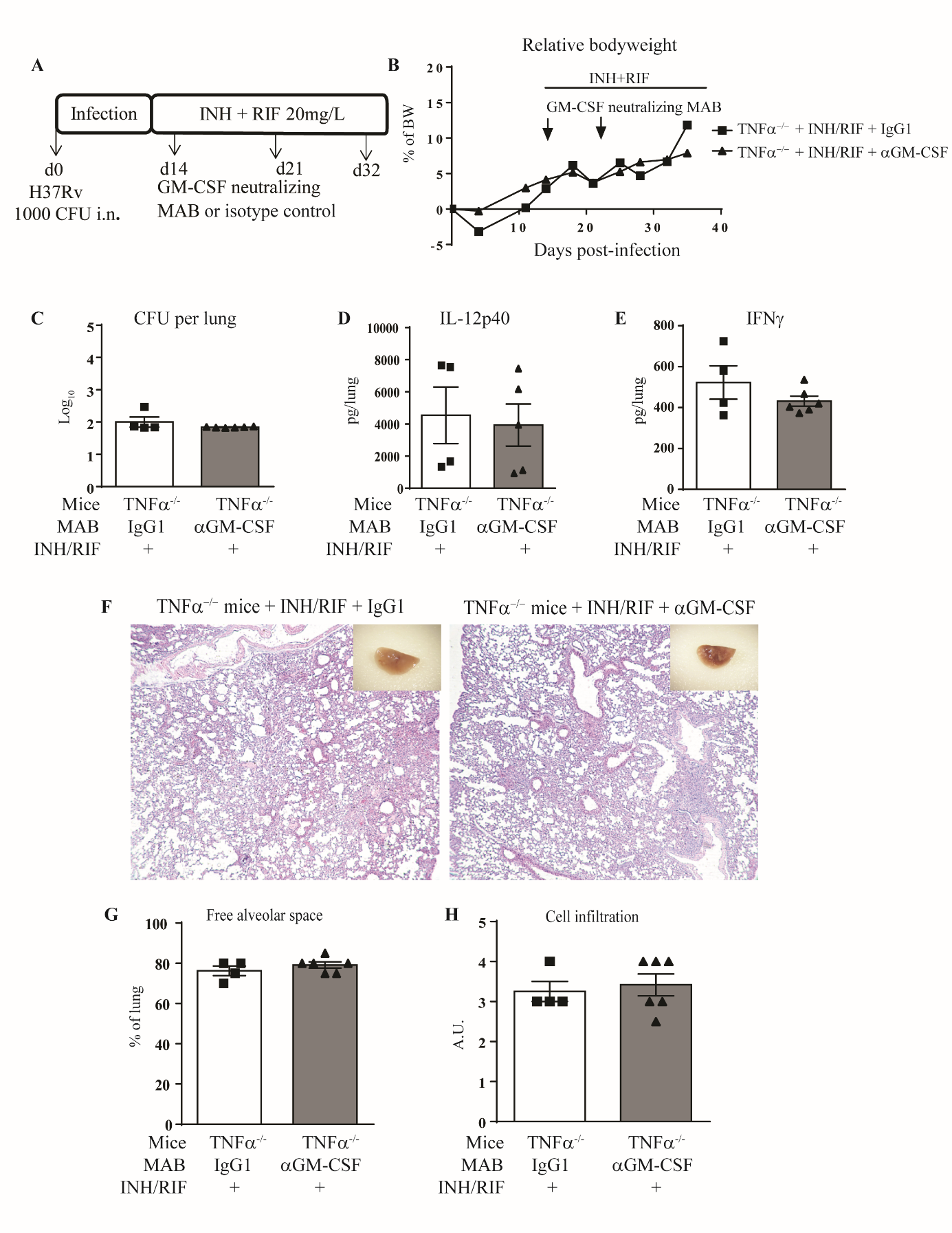
**

**Supplementary Figure 2. GM-CSF neutralization during control of *M. tuberculosis* infection by active chemotherapy**

TNFα^-/-^ mice were infected with *M. tuberculosis* H37Rv (1000±200CFU/mouse i.n.), treated with 20 mg/L of antibiotics (INH/RIF) in drinking water from day 14 to day 32 post-infection and GM-CSF was neutralized by anti GM-CSF MAB (Clone B2.6) or treated with IgG1 isotype control (A). The relative bodyweight (B) and lung bacterial burden (C) were measured after INH/RIF 20mg/L treatments on day 32 post-infection. IL-12p40 (D) and IFNγ (E) pulmonary levels were measured by ELISA. Lungs from infected mice were stained by HE (F) on day 32 post-infection and a scoring of free alveolar spaces (G) and cell infiltration (H) is shown. Data are expressed as mean ± SEM (n = 4-6 mice per group). No statistical difference between the groups were observed (p > 0.05).

**
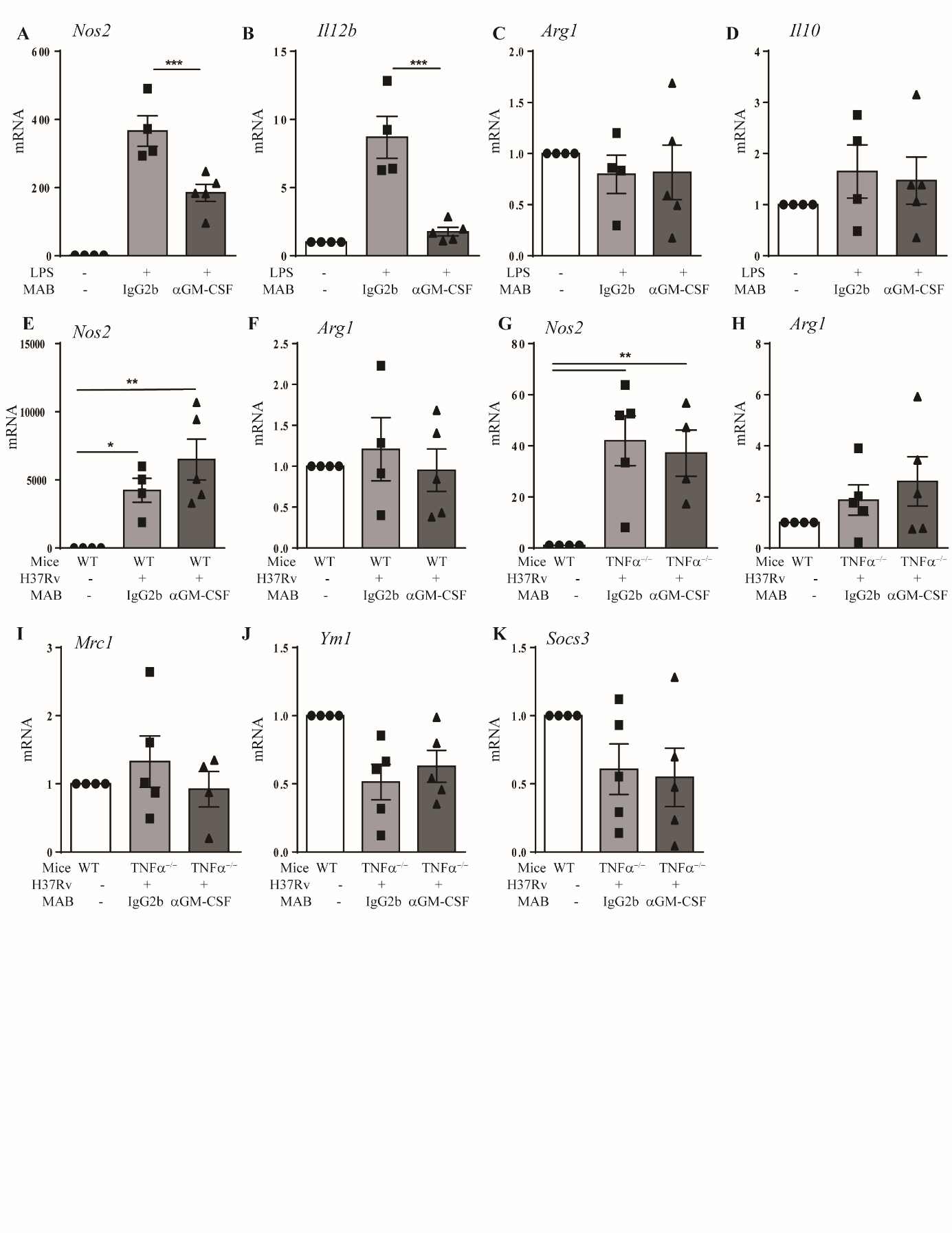
**

**Supplementary Figure 3. Limited impact of GM-CSF blockade on macrophage polarization after *M. tuberculosis* infection *in vivo***

WT mice were untreated or pre-treated with anti GM-CSF neutralizing antibody (200 µg Clone A7.39 per mouse i.p.) or IgG2b isotype control 1 hour prior to LPS aerogenic exposure (1 µg/mouse). After 24h, the lung mRNA levels of *nitric oxide synthase 2* (*Nos2*, A), *interleukin-12b* (*Il12b*, B), *arginase 1* (*Arg1*, C) and *interleukin-10* (*Il-10*, D) were analyzed by RT-qPCR. WT (E, F) and TNFα^-/-^ (G-K) mice were infected with *M. tuberculosis* and either untreated or treated with anti GM-CSF neutralizing antibody (200µg IgG2b i.p.) or isotype control, as in Figure 3. After 32 days for WT mice (E, F) and 26 days (G-K) post-infection for TNFα^-/-^ mice, the lung mRNA levels of *Nos2* (E, G), *Arg1* (F, H), CD206 *mannose receptor, C type 1* (*Mrc1*, I), *chitinase-like 3* (*Ym1*, J) and *suppressor of cytokine signaling 3* (*Socs3*, K) were analyzed by RT-qPCR. The fold change of mRNA levels, normalized to *Gapdh* expression level in uninfected mice, is shown. Data are expressed as mean ± SEM (n = 4-5 mice per group). Statistical comparisons are presented as compared to WT mice as a reference group (symbol above the bar in E-K) or in between groups treated with GM-CSF neutralizing MAB or isotype controls, as indicated. ***, p < 0.001, **, p < 0.01, *, p < 0.05.
